# Biogeographic distribution of autotrophic bacteria was more affected by precipitation than by soil properties in an arid area

**DOI:** 10.3389/fmicb.2023.1303469

**Published:** 2023-12-20

**Authors:** Ying Wang, Yimei Huang, Quanchao Zeng, Dong Liu, Shaoshan An

**Affiliations:** ^1^State Key Laboratory of Soil Erosion and Dryland Farming on the Loess Plateau, Northwest A & F University, Yangling, Shaanxi Province, China; ^2^College of Resources and Environment, Northwest A & F University, Yangling, Shaanxi Province, China; ^3^Key Laboratory of Horticultural Plant Biology, Ministry of Education, Huazhong Agricultural University, Wuhan, China; ^4^The Germplasm Bank of Wild Species, Yunnan Key Laboratory for Fungal Diversity and Green Development, Kunming Institute of Botany, Chinese Academy of Sciences, Kunming, China

**Keywords:** autotrophic bacteria, *cbbL* gene, *cbbM* gene, land use, precipitation

## Abstract

**Introduction:**

Autotrophic bacteria play an important role in carbon dioxide fixation and are widespread in terrestrial ecosystems. However, the biogeographic patterns of autotrophic bacteria and the driving factors still remain poorly understood.

**Methods:**

Herein, we conducted a 391-km north to south transect (mean annual precipitation <600 mm) survey in the Loess Plateau of China, to investigate the biogeographic distributions of autotrophic bacteria (RubisCO *cbbL* and *cbbM* genes) and the environmental drivers across different latitude sites with clear vegetational and climatic gradients.

**Results and discussion:**

The soils in northern region with lower precipitation are dominated by grassland/forest, which is typically separated from the soils in southern region with higher precipitation. The community structure of autotrophic bacterial *cbbL* and *cbbM* genes generally differed between the soils in the southern and northern Loess Plateau, suggesting that precipitation and its related land use practices/ecosystem types, rather than local soil properties, are more important in shaping the soil autotrophic microorganisms. The *cbbL*-containing generalist OTUs were almost equally abundant across the northern and southern Loess Plateau, while the *cbbM*-containing bacterial taxa were more prevalent in the low precipitation northern region. Such differences indicate differentiate distribution patterns of *cbbM*- and *cbbL*-containing bacteria across the north to south transect. Our results suggest that the community composition and the differentiate distributions of soil *cbbL*- and *cbbM*-containing bacterial communities depend on precipitation and the related ecosystem types in the north to south transect in the Loess Plateau of China.

## 1 Introduction

Carbon dioxide (CO_2_) fixation by autotrophic microorganisms is an important process in the carbon cycle of soil and contributes to soil organic C sequestration (Yuan et al., 2012a,b; Spohn et al., 2020). Autotrophic microorganisms in wetland and upland soils have similar potentials to the uptake of atmospheric CO_2_ (Long et al., 2015) and transfer it to soil organic C (Wu et al., 2014; Tang et al., 2015). Those microorganisms have been found in a wide variety of habitats, including arable soils, wetlands, freshwater, polluted water and oceans (Tolli and King, 2005; Yuan et al., 2012a; Alfreider et al., 2017). Thus, autotrophic microorganisms are ubiquitous in terrestrial ecosystems. Additionally, autotrophic bacteria also play critical roles in soil element cycling such as nitrogen and sulfur cycling (Aroca et al., 2007; Bazylinski et al., 2017). Therefore, autotrophic microorganisms may play important roles in regulating ecosystem functions. Yet, the biogeographic distribution of autotrophic microorganisms across different habitats and climatic gradients, and the environmental drivers remain largely unknown. Compared to the widely investigated biogeographic patterns of soil microorganisms with a taxonomic approach (Fierer, 2017; Bahram et al., 2018), a focus on microbial traits will have important implications in linking biogeographic diversity patterns and ecosystem processes (Green et al., 2008; Nelson et al., 2016).

Autotrophic microorganisms can fix atmospheric CO_2_ through six pathways, with the classical Calvin Benson Bassham (CBB) cycle being the most dominant and ubiquitous pathway in soils (Saini et al., 2011; Xiao et al., 2021). The Ribulose-1,5-bisphosphate carboxylase/oxygenase (RubisCO) is the main enzyme in the CBB cycle (Berg, 2011), exists in four forms (I, II, III and IV). These forms have different structures and catalytic activity (Tabita et al., 2007; Saini et al., 2011). Form IV is a homologous protein family of RubisCOs but lacking carboxylating activity; form III is a true RubisCO but does not confirm the occurrence of CBB cycle; only form I and II which occur in bacteria participate CO_2_ fixation through CBB pathway (Saini et al., 2011). Thus, the *cbbL* and *cbbM* genes, encoding the form I and II, respectively, are generally used to explore the diversity and ecology of autotrophic bacteria in environmental samples (Alfreider et al., 2012; Yuan et al., 2012b; Li et al., 2018). The occurrence of *cbbL* and *cbbM* genes varies among bacterial species, i.e., some bacteria have only *cbbL* gene, but some have both *cbbL* and *cbbM* genes (Shively et al., 1998; Saini et al., 2011). The *cbbL* gene is mainly affiliated with Proteobacteria, Actinobacteria, Firmicutes, Chloroflexi and Cyanobacteria (Tabita et al., 2007; Wang et al., 2021; Zhao et al., 2021). Form II (*cbbM*) is known only from Proteobacteria, markedly different from form I. Additionally, form II is alive in low O_2_ and high CO_2_ environments (Tabita, 1999; Badger and Bek, 2008), while the form I is believed to have evolved in response to the decline of CO_2_ and the emergence of oxygen as the Earth’s atmosphere changed (Elsaied and Naganuma, 2001). Alfreider et al. (2012) found that the distribution patterns and diversity of *cbbL* and *cbbM* genes in polluted groundwater were differentiate depending on redox conditions. While in other reports, both the *cbbL* and *cbbM* genes were present but no apparent different distribution pattern could be observed (Alfreider et al., 2017; Wang et al., 2022). These studies are mainly limited in water systems, suggesting that the distributions of form I and form II in environments and their driving factors still remain largely unclear.

Studies in soils mainly investigated the changes of autotrophic bacterial abundance and communities based on only *cbbL* gene in response to management practice and land use (Yuan et al., 2012a,b; Liu et al., 2016), focused on specific sites or local scale, and did not account for climate conditions. The changes of *cbbL*-containing autotrophic bacteria were generally related to organic C and its labile fractions in soils (Nanba et al., 2004; Yuan et al., 2012c). Climate conditions are important drivers of soil bacteria and fungi over large scales (Fierer, 2017; Bahram et al., 2018), and may also affect soil autotrophic microorganisms. Considering that the *cbbM* gene was also widely detected in environments in recent studies (Alfreider et al., 2009; Wang et al., 2021), it is necessary to study both the *cbbL* and *cbbM* genes for a systematic understanding of CO_2_-fixing microbial distribution and the driving factors. Zhao et al. (2018) found that mean annual precipitation was the main driver of soil autotrophic microbial abundance in desert, steppe and meadow soils in Tibetan Plateau. Aside from this study, limited information is available concerning the geographic patterns of soil autotrophic bacterial *cbbL* and *cbbM* genes and the driving factors in arid soils. Additionally, it remains largely unexplored whether climatic conditions, relative to local soil properties, is a dominant and/or equally important factor driving the distribution of soil autotrophic bacteria in arid soils at the large/landscape scale.

The Chinese Loess Plateau is widely considered as one of the most severely eroded regions in the world, driving the implementation of the “Grain for Green” Program (GTGP) since 1999 (Chen et al., 2015). Such program substantially resulted in land-use conversions from agriculture to forest, shrub and grassland (Deng et al., 2012), which is important in soil organic C storage (Wiesmeier et al., 2019). Carbon input to soils by plants experiences different turnover processes from that by autotrophic microorganisms. The organic C by plant inputs is mineralized and lost through CO_2_, with only a small portion (<5%) contributed to soil organic C storage (Liu et al., 2019). Inputs of fresh organic C to soil can also stimulate microbial activities, leading to a priming effect of old soil organic C (Luo et al., 2016). Carbon fixed by autotrophic microorganisms is expected to be more stable in soil than plant residues, and contributes a significant fraction to the stable soil organic C pools (Spohn et al., 2020; Xiao et al., 2021). However, a systematic study of autotrophic bacteria in the Chinese Loess Plateau is still lacking. Such information might be of importance in understanding current and future soil C cycling. Here, we focused on the north to south transect of the Loess Plateau, which has great gradients in climatic and vegetational and geographic distance variables. We investigated the distribution of *cbbL*- and *cbbM-*containing bacteria by high-throughput sequencing and explored the environmental drivers such as soil parameters, vegetation, climate and geographic distance. We hypothesized that the varied climatic and vegetational conditions across the north to south regions in the Chinese Loess Plateau would dramatically alter the community structure of soil autotrophic bacteria and differentially enrich *cbbL*- and *cbbM-*containing bacterial taxa.

## 2 Materials and methods

### 2.1 Site description and soil sampling

The Chinese Loess Plateau is one of the most important serious erosion areas, including forest, grassland, shrub land and cropland ecosystems. In this study, we collected 24 soil samples from a north–south transect across the Chinese Loess Plateau (Supplementary Figure S1), which represented four types of ecosystems (grassland to dessert grassland, forest-grassland, forest and agriculture) and a wide range of climatic conditions (mean annual temperature (MAT) ranged from 8.7°C to 13.1°C, and mean annual precipitation (MAP) ranged from 371 mm to 585 mm). Soils in the study area are mainly derived from loess and further classified as Calcic Cambisols (IUSS Working group WRB, 2014) with silty loam. The 8 sampling sites in the north–south transect included Jingbian (JB) (forest steppes), Liandaowan (LDW) (grassland), Ansai (AS) (forest steppes), Ziwuling (ZWL) (mountain forests), Luochuan (LC) (orchard), Weibei (WB) (orchard) and Guanzhong (GZ) (agriculture), and Qinling (QL) (mountain forest). The codes of A, B and C following the above site abbreviations represent various biological replicates at landscape scales. In each site, three replicate subplots were randomly selected with an area of 15 m × 15 m. Six cores (0–10 cm depth, 2 cm diameter) were taken from each subplot and mixed to form one composite sample, i.e., three replicates for each site. The distances between soil sampling sites ranged from 0.3 km to 391 km (Liu et al., 2018). After removing stones and visible plant residues, soil samples were sieved through a 2-mm mesh, then a 10 g subsample from each plot was immediately wrapped in aluminum foil, quenched with liquid N_2_, and stored at −80°C until the extraction of soil DNA. The MAP and MAT data were collected from the Chinese meteorological database.^1^ The geographical characteristics, typical vegetation and climatic factors of the sampling sites were shown in Table 1. The soil physicochemical parameters used in this study were recently published (Liu et al., 2018).

**Table 1:** Soil sampling site characteristics.

Sample	Vegetation†	Ecosystem type	Latitude† (N)	Longitude† (E)	MAP (mm)	MAT (°C)
JBA	*Salix cheilophila*	Forest steppes	37°31′20.21′′	108°51′38.41′′	372	8.7
JBB	*Salix matsudana Koidz*	Forest steppes	37°30′9.63′′	108°52′58.06′′	373	8.8
JBC	*Populus simonii Carr*	Forest steppes	37°28′16.91′′	108°54′8.08′′	378	8.8
LDWA	*Bothriochloa ischaemum L.*	Grassland	37°11′39′′	108°58′15′′	417	9.0
LDWB	*Artemisia scoparia Waldst*	Grassland	37°10′12′′	108°57′01′′	417	9.0
LDWC	*Stipa grandis*	Grassland	37°11′49.06′′	108°57′11.34′′	413	9.0
ASA	*Artemisia gmelinii*	Forest steppes	36°47′23.30′′	109°16′32.67′′	477	9.4
ASB	*Caragana Korshinskii*	Forest steppes	36°43′54.22′′	109°15′29.74′′	482	9.4
ASC	*Robinia pseudoacacia*	Forest steppes	36°43′56.05′′	109°15′18.10′′	482	9.4
ZWLA	*Pinus tabuliformis*	Mountain forest	36°0′18.15′′	109°5′54.50′′	538	9.2
ZWLB	*Carrière*	Mountain forest	36°1′42′′	108°52′43′′	537	9.1
ZWLC	*Betula platyphylla*	Mountain forest	36°5′56.70′′	108°37′28.56′′	524	8.9
LCA	*Malus pumila Mill*	Orchard/Agriculture	35°47′43.77′′	109°28′31.76′′	543	9.7
LCB	*Malus pumila Mill*	Orchard/Agriculture	35°49′30.47′′	109°29′0.83′′	541	9.7
LCC	*Malus pumila Mill*	Orchard/Agriculture	35°50′32.16′′	109°24′28.55′′	542	9.6
WBA	*Amygdalus persica L.*	Orchard/Agriculture	34°43′55.22′′	109°13′45.74′′	541	12.7
WBB	*Pyrus spp*	Orchard/Agriculture	34°54′38.12′′	109°36′4.50′′	528	12.5
WBC	*Malus pumila Mill*	Orchard/Agriculture	35°11′45.60′′	107°48′26.13′′	567	9.7
GZA	*Zea mays L.*	Agriculture	34°25′59.98′′	107°39′45.96′′	584	11.3
GZB	*Zea mays L.*	Agriculture	34°20′28.46′′	108°0′41.66′′	563	12.2
GZC	*Zea mays L.*	Agriculture	34°18′15.43′′	108°22′53.05′′	539	12.9
QLA	*Quercus wutaishanica*	Mountain forest	34°6′25.74′′	108°11′36.7′′	559	13.2
QLB	*Quercus acutidentata*	Mountain forest	34°10′52.14′′	107°39′2.15′′	583	12.5
QLC	*Quercus wutaishanica*	Mountain forest	34°4′15.23′′	108°1′5.27′′	575	13.1

†The information derived from Liu et al. (2018).

### 2.2 DNA extraction and Illumina MiSeq sequencing

The same soil DNA samples of the bacterial community analysis (Liu et al., 2018) were used in this study. Briefly, the soil DNA was extracted by the MoBio Power Soil DNA isolation kit (12888) according to the manufacturer’s instructions. The purified soil DNA was checked for quality and quantity using a NanoDrop Spectrophotometer. Then, the PCR amplification was performed for *cbbL* gene (K2f: 5’-ACCAYCAAGCCSAAGCTSGG-3′; V2r: 5’-GCCTTCSAGCTTGCCSACCRC-3′) (Nanba et al., 2004) and *cbbM* gene (*cbbM*-F: 5’-TTCTGGCTGGGBGGHGAYTTYATYAARAAYGACGA-3′; *cbbM*-R: 5’-CCGTGRCCRGCVCGRTGGTARTG-3′) (Campbell and Cary, 2004), respectively. PCR was performed in a 25 μL volume mixture containing 5 μL reaction buffer (5×), 5 μL GC buffer (5×), 2 μL dNTP (2.5 mM), 1 μL forward primer (10 μM), 1 μL reverse primer (10 μM), 2 μL DNA template, 8.75 μL ddH_2_O, and 0.25 μL Q5 DNA Polymerase (NEB). Primers were tagged with sample-specific 7-bp barcodes for multiplex sequencing. Negative controls using sterilized water instead of soil DNA were included to check for primer or sample DNA contamination. The following thermal program was used for amplification: initial denaturation at 98°C for 2 min, followed by 30 cycles at 98°C for 15 s, 55°C (*cbbL*) or 57°C (*cbbM*) for 30 s, 72°C for 30 s, with a final extension at 72°C for 10 min. Each DNA sample was amplified in three technical replicates and then verified with electrophoresis and mixed in one tube. All samples were purified with the agarose gel DNA Recovery Kit and pooled together with equal molar amounts from each sample, then sequenced using the Illumina Miseq platform (Illumina Inc., San Diego, CA, USA).

The raw sequence data were first merged using Vsearch’s fastq_mergepairs module, followed by quality filtered and dereplicated using fastq_filter and derep_fullength modules. The low-quality sequences with length < 150 bp, ambiguous bases in barcodes and mononucleotide repeats >8 bp were filtered out. Then, the sequences were used for a chimera check via uchime_denovo module; the non-chimera sequences were clustered at 97% by cluster_size module to generate operational taxonomic units (OTUs) representative sequences and OTUs table. Sequence alignment was performed using the localized nucleotide sequence database.^2^ The representative sequences for each OTU were assigned to taxonomic groups by BLAST searching the representative sequences set against the NCBI nucleotide sequence database^3^ using the best hit. The original sequence data are archived at the European Nucleotide Archive (ENA) with accession number PRJEB58633.^4^

### 2.3 Data analysis

Each sample was rarefied to the same number of reads (10,369 reads for *cbbL* and 17,000 reads for *cbbM*) for OTU level alpha-diversity and beta-diversity metrics. The index included Chao1 estimator of richness, observed species, evenness, shannon’s diversity index and Bray-Curtis distance between samples. The 24 sampling sites were separated into northern (including JB, LDW, AS and ZWL with 3 biological replicates) and southern (including LC, WB, GZ and QL with 3 biological replicates) regions. The OTUs occurring in at least 9 of 12 samples in the northern or southern region and 20 of 24 samples in the Loess Plateau were defined as generalists.

Principal component analysis (PCA) was calculated using soil environment parameters and climatic parameters as variables. The significant difference of soil variables and autotrophic bacterial groups between the northern and southern regions was tested by nonparametric one-way analysis using Kruskal-Wallis test. The simple linear regression analysis (SPSS 18.0 for Windows) was used to test the relationships between geochemical features and soil autotrophic bacterial taxa proportion. The rate of distance-decay of the autotrophic bacterial communities was calculated as the slope of a linear regression on the relationship between the geographic distance and the bacterial similarity based on 1-dissimilarity of the Bray-Curtis metric. Canonical correspondence analysis (CCA) was used to identify the abiotic factors (soil, climate and geographic coordinates) that are significantly related/contributed to soil autotrophic bacterial communities by Canoco 5.0. Significant differences in microbial community structure between soils in the northern and southern regions were determined by multi-response permutation procedures (MRPP) (*PC-ORD* 5.0, MjM software, www.pcord.com).

## 3 Results

### 3.1 Geochemical parameters in the Loess Plateau

**Figure 1:**
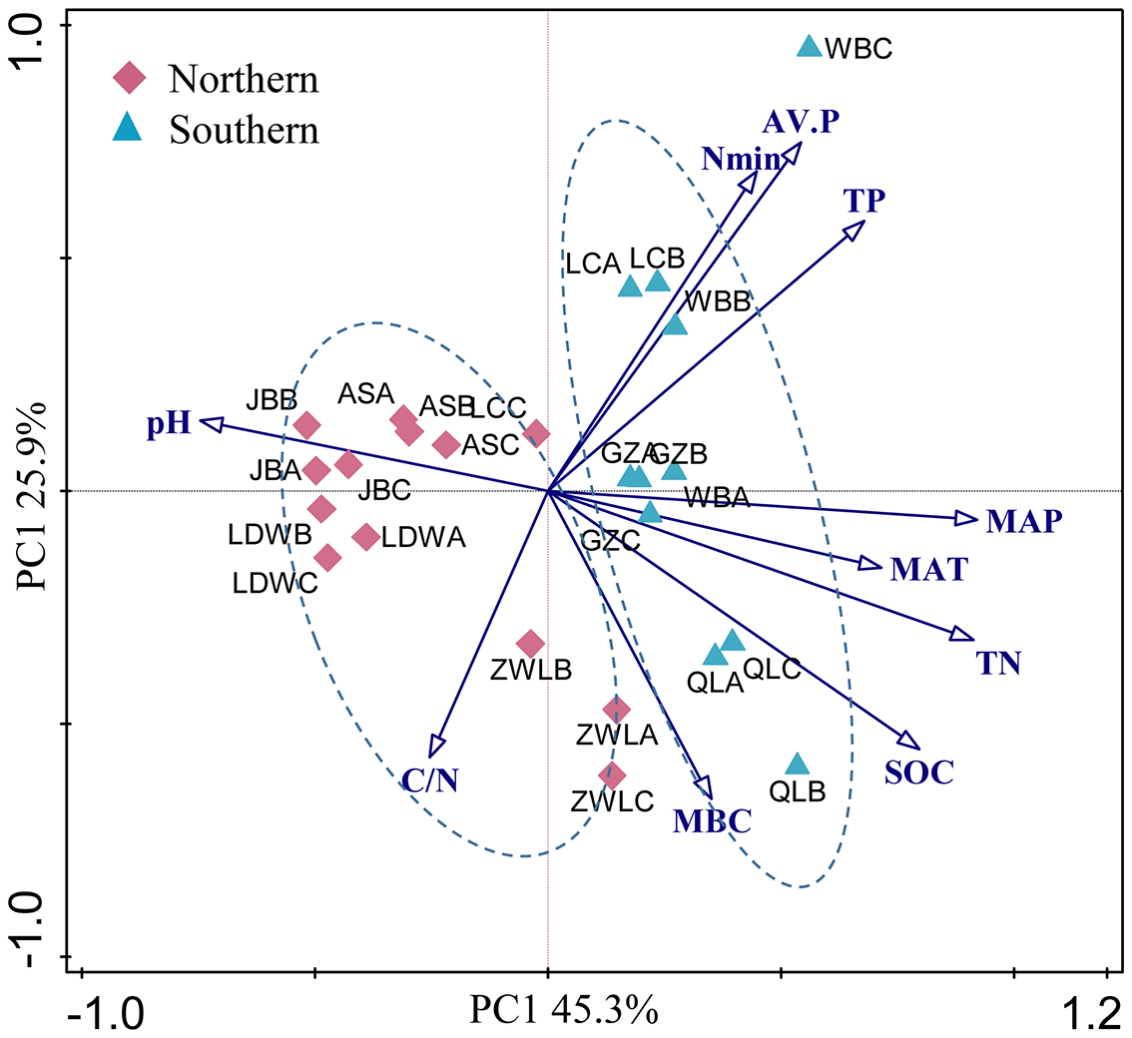
(A) Principal component analysis (PCA) based on soil physicochemical characteristics and climatic parameters as variables. MAP: mean annual precipitation; MAT: mean annual temperature; SOC, soil organic C; TN, soil total N; C/N: the ratio of SOC to TN; TP: soil total P; AV.P: available P; Nmin: the sum of ammonium and nitrate; MBC: microbial biomass C. JB: Jingbian; LDW: Liandaowan; AS: Ansai; ZWL: Ziwuling; LC: Luochuan; WB: Weibei; GZ: Guanzhong; QL: Qinling, with 3 replicates (A, B, C), respectively.

Soil geographic and chemical parameters were described in our previous study (Table 1, Liu et al., 2018). Briefly, the concentrations of soil organic C, total N and total P ranged from 2.6 to 21.6 g kg^−1^, 0.2–2.0 g kg^−1^ and 0.26–1.5 g kg^−1^, respectively. In addition, soil available N and P concentrations were 8.9–269 g kg^−1^ and 0.4–87.2 g kg^−1^, respectively. These soil parameters generally showed strongly positive correlation with MAP and MAT, and negative correlation with latitude (Supplementary Table S1). In contrast, soil pH was significantly and negatively correlated with MAP and MAT. PCA analysis of soil and climatic parameters showed that soil samples from WB, LC, GZ and QL clustered together (Figure 1). These soils were generally separated from soils in JB, LDW, AS and ZWL in PC1, indicating their large environmental heterogeneity. MAP and MAT were the main factors contributing to the large environmental heterogeneity. The soil sampling sites were separated into northern (JB, LDW, AS and ZWL) and southern (WB, LC, GZ and QL) regions. The northern region was dominated by forest/grassland ecosystems, and the southern region was dominated by agriculture except QL (forest) (Table 1). MAP, MAT and soil parameters except MBC and C/N ratio were significantly different between the northern and southern regions (Supplementary Table S2).

### 3.2 Taxonomic diversity

High throughput sequencing analysis of the Loess Plateau soil samples revealed 15,604 and 2,781 species-level OTUs for the *cbbL* and *cbbM* genes, respectively. The Chao1 richness and Shannon diversity of the *cbbL* and *cbbM* genes showed no difference between the northern and southern regions (Supplementary Table S3). For the *cbbL* gene, Proteobacteria- and Actinobacteria-like sequences were predominant, accounting for 43.5–88.9% and 8.0–35.7% of the total reads, respectively; Cyanobacteria- and Firmicutes-like sequences were also detected in most soils in low relative abundance (< 0.13%). The phylum Proteobacteria was represented by classes Alphaproteobacteria (21.0–53.4%), Betaproteobacteria (4.8–40.5%), Gammaproteobacteria (0.5–37.9%) and Deltaproteobacteria (0–0.2%) (Supplementary Figure S2A). For the *cbbM* gene, phylum Proteobacteria was predominant and accounted for 34–99% of the total reads (Supplementary Figure S2B), and represented by classes Alphaproteobacteria (0.3–81.7%), Betaproteobacteria (2.1–84.0%), Gammaproteobacteria (3.0–72.2%) and Acidithiobacillia (0.4–66.0%) (Supplementary Figure S2B).

**Figure 2:**
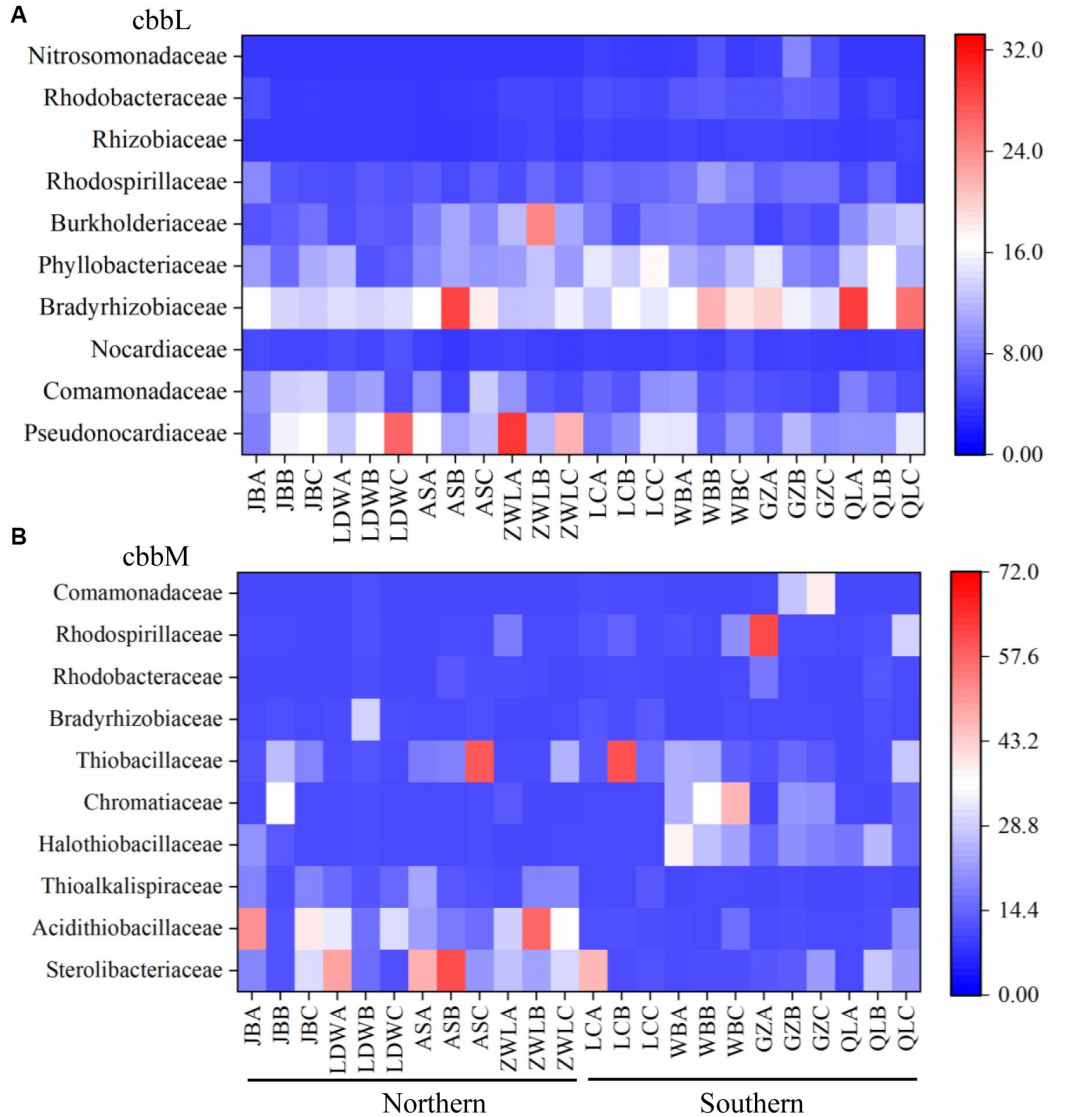
The top 10 families of autotrophic bacteria in the soils along a north–south transect in the Chinese Loess Plateau. **(A)** *cbbL*-containing bacteria; **(B)** *cbbM*-containing bacteria. JB: Jingbian; LDW: Liandaowan; AS: Ansai; ZWL: Ziwuling; LC: Luochuan; WB: Weibei; GZ: Guanzhong; QL: Qinling, with 3 replicates (A, B, C), respectively.

The changes of *cbbL* and *cbbM* genes across the Chinese Loess Plateau were also explored at family level (Figure 2, Supplementary Table S3). For *cbbL* communities, the family Pseudonocardiaceae was higher in the northern than in the southern Loess Plateau, while Bradyrhizobiaceae, Phyllobacteriaceae, Rhodospirillaceae, Rhizobiaceae and Rhodobacteraceae were higher in the southern Loess Plateau (Figure 2A, Supplementary Table S3). The family Nitrosomonadaceae was only detected in the southern Loess Plateau. For *cbbM* communities, the families Sterolibacteriaceae, Acidithiobacillaceae and Thioalkalispiraceae were higher in the northern than in the southern Loess Plateau, while Halothiobacillaceae, Rhodospirillaceae and Comamonadaceae were higher in the southern Loess Plateau (Figure 2B, Supplementary Table S3). The *cbbM* gene identified as family Comamonadaceae was mainly composed with *Limnohabitans*-like sequences; the *cbbL* gene in this family was dominated by Var*iovorax*-like sequences.

### 3.3 Predictors of regional community structure

**Figure 3:**
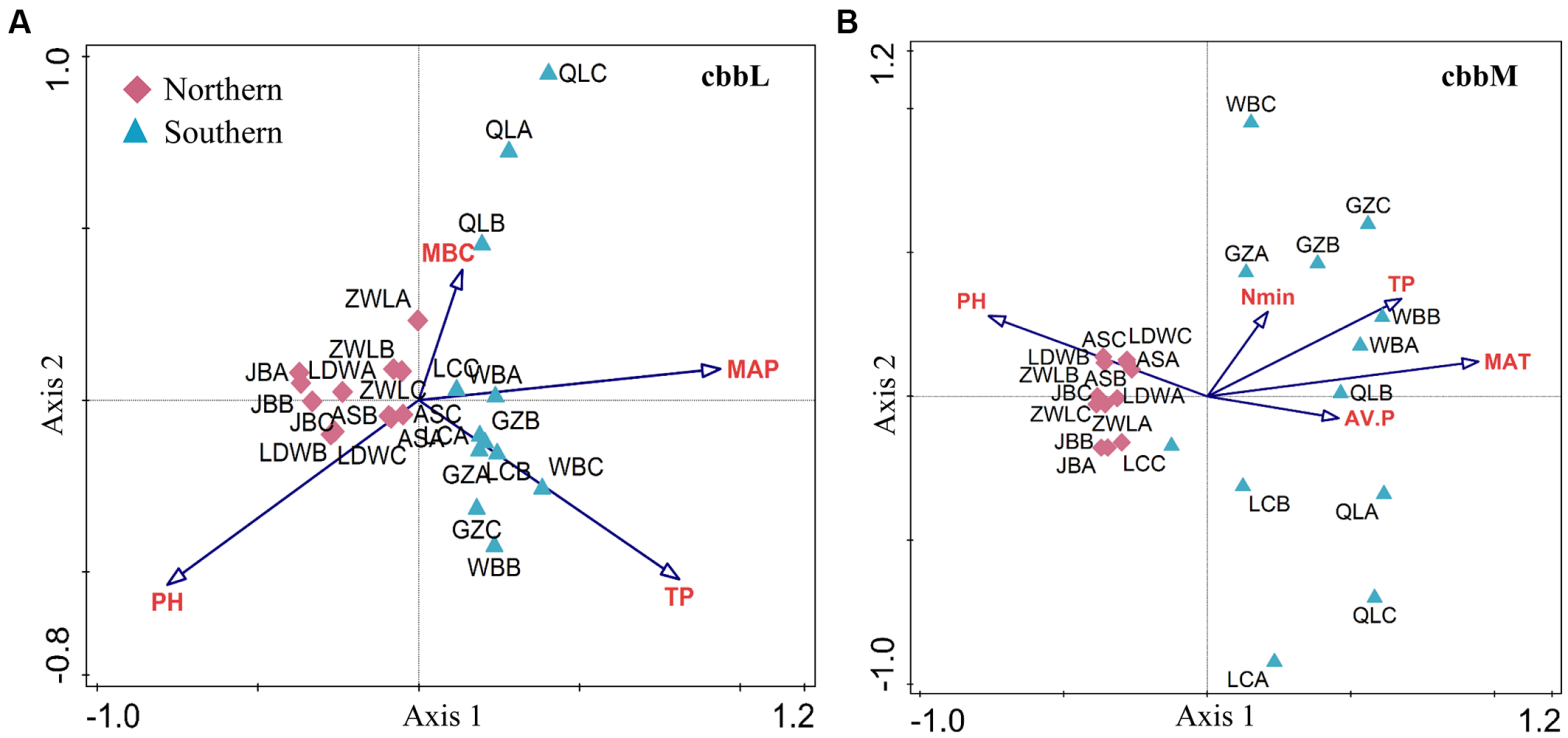
Factors driving the β-diversity of soil *cbbL*- and *cbbM*-containing bacterial communities in the Chinese Loess Plateau. The *cbbL*- **(A)** and *cbbM*-containing bacteria **(B)** in the Chinese Loess Plateau. MAP: mean annual precipitation; MAT: mean annual temperature; TP: soil total P; AV.P: available P; MBC: microbial biomass C. JB: Jingbian; LDW: Liandaowan; AS: Ansai; ZWL: Ziwuling; LC: Luochuan; WB: Weibei; GZ: Guanzhong; QL: Qinling, with 3 replicates (A, B, C), respectively.

The *cbbL-* and *cbbM-*containing bacterial community structure in soils from JB, AS, LDW and ZWL was clustered together, which was separated from that in WB, LC, GZ and QL soils in PC1 (Figures 3A,B). Such difference was confirmed by the MRPP analysis (Supplementary Table S4). CCA analysis demonstrated that soil pH, total P and MBC and MAP significantly affected the *cbbL-*containing bacterial community structure (Figure 3A). While soil pH, available P, total P and mineral N and MAT were significantly related to *cbbM-*containing bacterial community structure (Figure 3B). Region-specific predictors of autotrophic bacterial communities were explored for soil samples in the northern and southern regions, respectively. In the northern region, soil pH, MBC and TP significantly affected the *cbbL-*containing bacterial community structure, while pH, TP and available P significantly affected the *cbbM-*containing bacterial community structure (Supplementary Figures S3A,B). Soil pH, TP and MAT had stronger influences on the changes of *cbbL-* and *cbbM-*containing bacterial communities in the southern region (Supplementary Figures S3C,D).

For *cbbL* gene, Pseudonocardiaceae was negatively correlated with soil TP, while Phyllobacteriaceae, Rhizobiaceae Rhodobacteraceae and Nitrosomonadaceae were generally positively correlated with soil TP and negatively correlated with soil pH (Table 2). For *cbbM* gene, Sterolibacteriaceae, Acidithiobacillaceae and Thioalkalispiraceae were generally positively correlated with soil pH and negatively correlated with soil TP; Halothiobacillaceae and Rhodospirillaceae were negatively correlated with soil pH and generally positively correlated with soil TN and TP (Table 2). In the northern region, Pseudonocardiaceae and Rhizobiaceae of *cbbL* gene were generally negatively correlated with soil pH and positively correlated with soil organic C and TN; Halothiobacillaceae and Bradyrhizobiaceae of *cbbM* gene were positively correlated with soil pH and negatively correlated with soil TN or MBC (Supplementary Tables S5,6). In the southern region, Bradyrhizobiaceae and Burkholderiaceae of *cbbL* gene were generally negatively correlated with soil pH and positively correlated with soil TN; Rhodospirillaceae, Rhodobacteraceae and Nitrosomonadaceae of *cbbL* gene were positively correlated with soil TP. Comamonadaceae-like sequences of *cbbM* gene were positively corrected with soil pH.

**Table 2:** Spearman correlations of soil chemical and microbial variables with *cbbL*-containing bacterial families.

Communities	MAT	MAP	pH	MBC	TN	TP	SOC
*cbbL*							
Pseudonocardiaceae	−0.402	−0.357	0.261	−0.022	−0.070	−0.459*	−0.043
Comamonadaceae	−0.386	−0.419*	0.289	−0.243	−0.358	−0.443*	−0.413*
Bradyrhizobiaceae	0.371	0.266	−0.296	−0.003	0.147	0.188	0.063
Phyllobacteriaceae	0.327	0.573**	−0.523**	0.279	0.403	0.277	0.265
Burkholderiaceae	−0.024	0.103	−0.207	0.387	0.317	−0.228	0.417*
Rhodospirillaceae	0.216	0.200	−0.193	0.112	0.051	0.544**	0.073
Nocardiaceae	−0.285	−0.292	0.200	−0.488*	−0.310	−0.197	−0.437*
Rhizobiaceae	0.465*	0.677**	−0.534**	0.363	0.573**	0.599**	0.537**
Rhodobacteraceae	0.431*	0.473*	−0.417*	0.369	0.372	0.714**	0.382
Nitrosomonadaceae	0.720**	0.637**	−0.563**	0.101	0.366	0.816**	0.323
							
*cbbM*							
Sterolibacteriaceae	−0.435*	−0.312	0.400	−0.039	−0.235	−0.458*	−0.145
Acidithiobacillaceae	−0.736**	−0.565**	0.484*	−0.134	−0.301	−0.643**	−0.290
Thioalkalispiraceae	−0.846**	−0.698**	0.666**	−0.157	−0.482*	−0.659**	−0.451*
Halothiobacillaceae	0.571**	0.372	−0.419*	0.003	0.221	0.465*	0.194
Chromatiaceae	0.177	−0.058	−0.110	−0.329	−0.107	0.259	−0.141
Thiobacillaceae	−0.134	−0.167	0.088	−0.195	−0.105	0.044	−0.190
Bradyrhizobiaceae	−0.241	−0.068	0.288	−0.357	−0.323	−0.097	−0.497*
Rhodobacteraceae	−0.106	0.235	0.081	0.160	0.046	−0.168	−0.090
Rhodospirillaceae	0.470*	0.694**	−0.583**	0.332	0.605**	0.563**	0.439*
Comamonadaceae	0.520**	0.545**	−0.327	0.279	0.287	0.446*	0.340

* *p* < 0.05 level.

** *p* < 0.01 level.

MAT: mean annual temperature; MAP: mean annual precipitation; SOC: soil organic C; TN: total N; TP: total P; MBC: microbial biomass C.

### 3.4 Macroecological patterns

**Figure 4:**
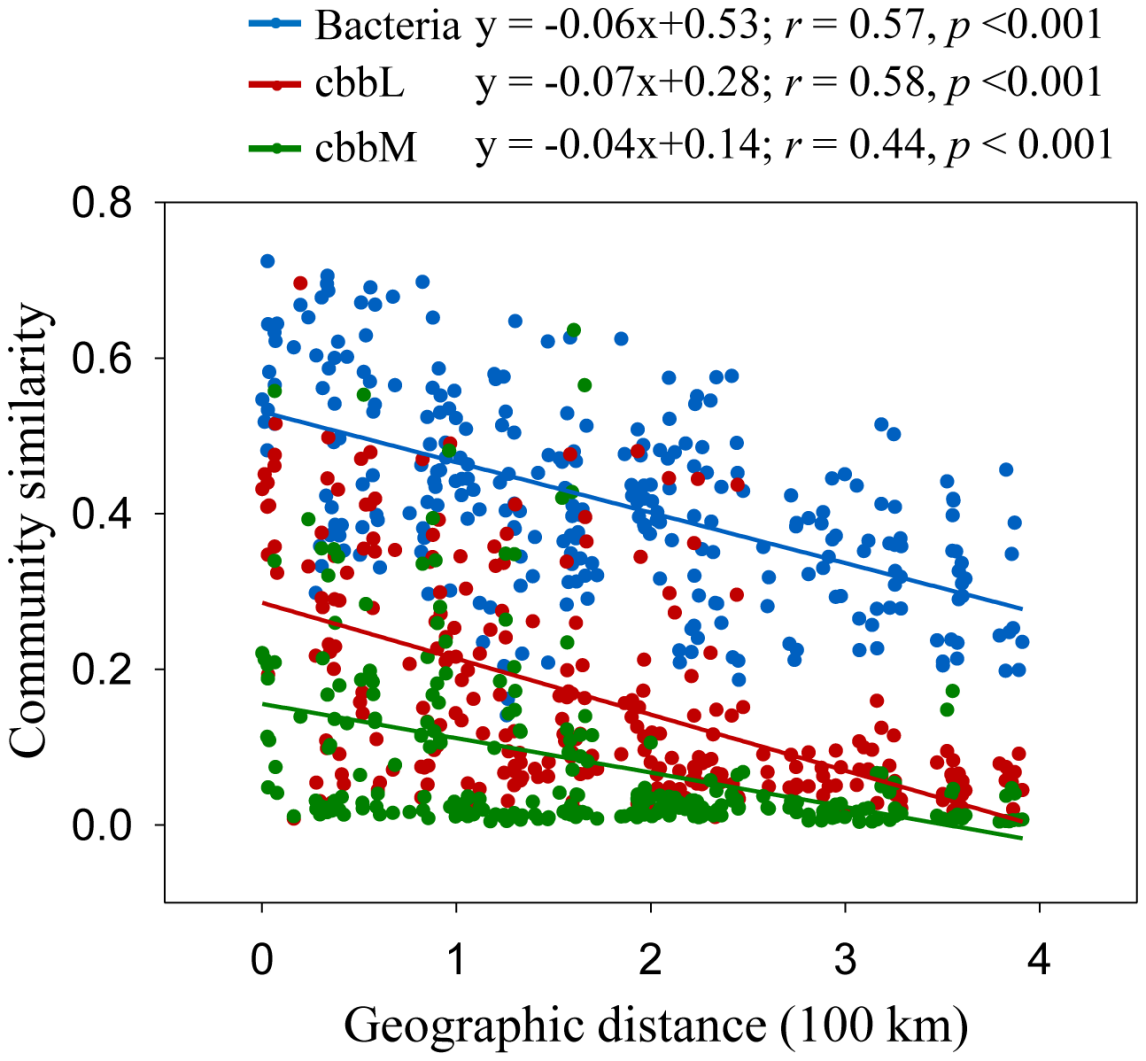
Correlation between community dissimilarities (Bray-Curtis distance) and geographic distances. Bray-Curtis distance of bacterial OTUs were calculated from Liu et al. (2018).

**Figure 5:**
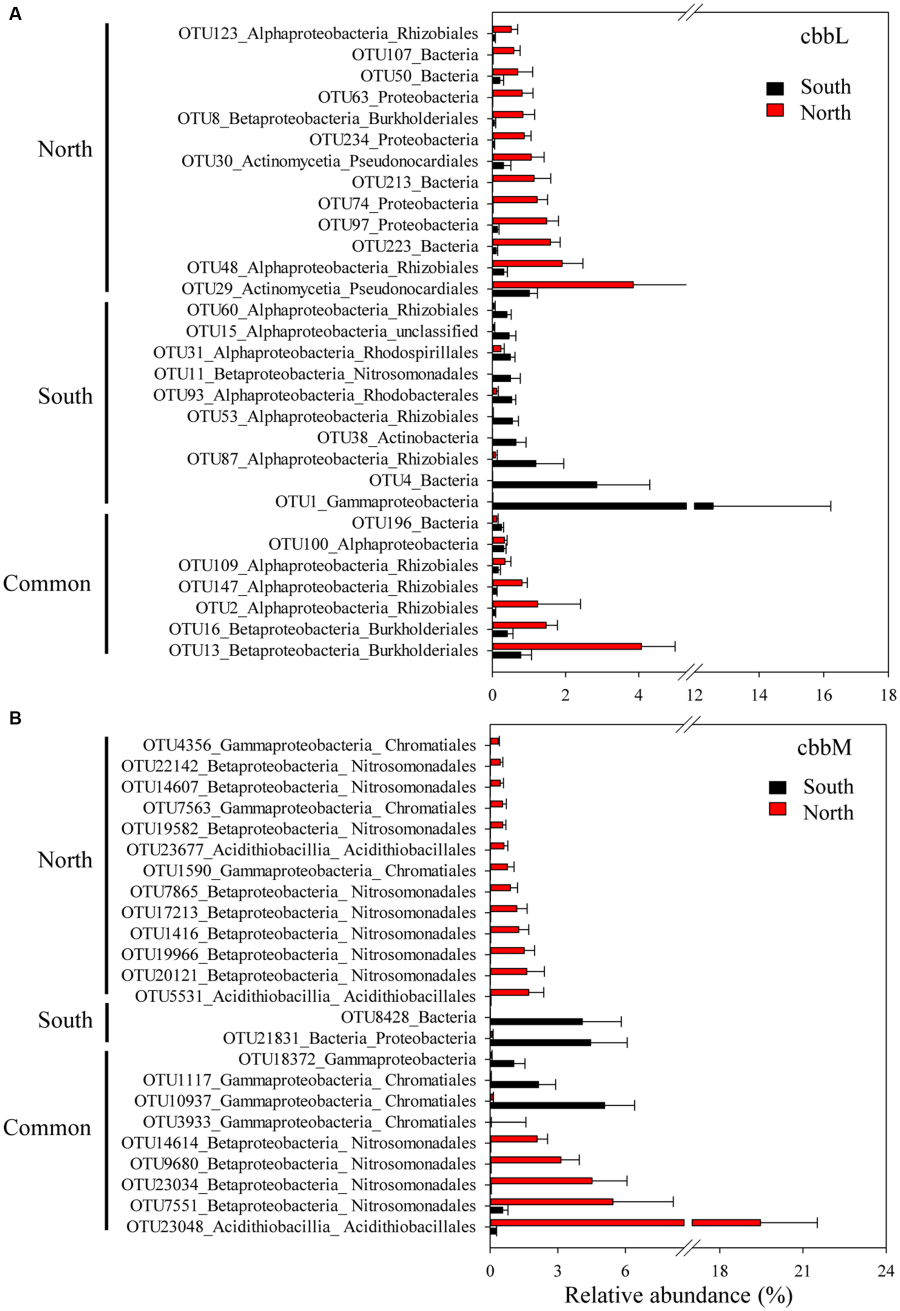
Generalist OTUs distribution in the northern and southern Chinese Loess Plateau. **(A)** *cbbL*-containing bacteria, **(B)** *cbbM*-containing bacteria.

The importance of spatial distance in autotrophic bacterial community variability was estimated based on Bray-Curtis distance, which revealed the distance-decay relationships of community similarity vs. geographic distance for each pairwise set of samples for *cbbL* or *cbbM* gene (Figure 4). To further investigate the similarity of autotrophic bacterial distribution in the Loess Plateau soils, the broadly distributed autotrophic bacterial taxa were described. A total of 7 (*cbbL*) and 9 (*cbbM*) OTUs present in the Loess Plateau soils were identified and defined as generalist (Figure 5), that accounted for 0.5–2.5% and 2.0–7.2% of the total OTUs in each sample, respectively, but contributed to a higher proportion of the total reads (0.1–19.1% for *cbbL*, 0.3–50.7% for *cbbM*). The generalist OTUs were also identified for the soils in the northern and southern regions, respectively. The compositions of *cbbL-* and *cbbM-*containing bacterial generalist OTUs differed greatly between the southern and northern regions (Figure 5). Moreover, the *cbbL-*containing generalist OTUs were almost equally abundant in the southern and northern regions. Whilst *cbbM-*containing bacterial taxa were more prevalent and showed a higher abundance in the northern region compared to the southern region.

## 4 Discussion

### 4.1 Autotrophic bacterial community shaped by precipitation and ecosystem types in the Loess Plateau

In this study, autotrophic bacterial communities were investigated in soils with great climatic and vegetational gradients in the Chinese Loess Plateau. In general, precipitation increases from the north to south transect, with the northern region dominated by grassland/forest soils and the southern region dominated by agricultural soils except QL which has the highest MAP and is represented by mountain forest. The *cbbL*- and *cbbM-*containing bacterial communities in the southern soils including QL were generally separated from that in the northern grassland/forest soils (Figure 3, Supplementary Table S4), highlighting the important role of MAP and its related land use practices/ecosystem types on shaping soil autotrophic bacteria. Additionally, the compositions of *cbbL-* and *cbbM-*containing bacterial generalist OTUs differed greatly between the southern and northern regions (Figure 5). However, our recent study in the same soils found that soil bacteria was strongly influenced by soil pH, followed by soil TP and TN (Liu et al., 2018), rather than MAP and its related land use practices/ecosystem types. Other large-scale studies also showed that bacterial community structure varies strongly with soil and climate variables: Bahram et al. (2018) found that the composition of global topsoil bacterial communities responded most strongly to soil pH, followed by precipitation; Liao et al. (2022) found that soil *cbbL-*containing bacteria was significantly correlated with soil pH and climate factors (MAP and MAT). Such different distribution patterns might be caused by two reasons. First, the natural ecosystems and precipitation in the northern Loess Plateau might shape a different niche for autotrophic bacteria compared to the southern Loess Plateau. This is partly supported by a previous study which found a significant role of precipitation changes in affecting *cbbL-*containing bacterial communities in a grassland soil (Li et al., 2022). Second, the relatively low local soil pH ranges in this study, i.e., 8.3–9.1 in most soils, might show a less important role on altering the overall soil autotrophic bacterial community (Bahram et al., 2018). Thus, there is a differentiation in distribution and environmental drivers of total bacteria and autotrophic bacteria in the north–south transect with great differences in vegetation, climatic and geographic distance in the Chinese Loess Plateau.

The different ecosystem types and precipitation in the northern and southern regions and their impact on soil pH and nutrient concentrations such as TN, TP and soil organic C changed autotrophic bacteria at family level. For instance, Rhodospirillaceae and Rhodobacteraceae of *cbbL* gene were higher in the southern Loess Plateau and positively correlated with soil TP; Nitrosomonadaceae was only detected in the southern Loess Plateau and positively correlated with soil pH and TP and negatively correlated with TN. In contrast, several families of *cbbM* gene were dependent on MAP and ecosystem types but were related to the differences in soil properties in the whole Loess Plateau. For instance, Sterolibacteriaceae, Acidithiobacillaceae and Thioalkalispiraceae were dominant families and generally higher in the northern than in the southern Loess Plateau, but only positively correlated with soil pH and negatively correlated with soil TP in the whole Loess Plateau. The results suggest that these taxa might be sensitive to the differences in soil pH, in agreement with previous findings that soil pH is a robust and useful predictor of bacterial communities in global and local scale studies (Zhou et al., 2015; Fierer, 2017). *CbbL-*containing bacterial abundance and diversity have been shown to relate to soil organic C labile fractions (DOC and MBC) and soil N concentrations (Lynn et al., 2017; Wang et al., 2022). The c*bbM-*containing bacteria are less studied compared to the *cbbL-*containing bacteria. Soil phosphorus is also an important driver for autotrophic bacteria in the Chinese Loess Plateau. However, knowledge about the effect of soil phosphorus on autotrophic microorganisms is limited (Yuan et al., 2015). The physiological regulators of autotrophic communities require further investigation under a range of P conditions to understand their community dynamics under different ecosystem types.

We also explored the distributions of generalist OTUs of autotrophic bacteria in the northern and southern Loess Plateau. The *cbbL-*containing generalist OTUs showed almost equal abundance in the southern and northern regions, while *cbbM-*containing bacterial taxa were more prevalent in the northern region with grassland/forest (Figure 5), suggesting their different dependency on MAP and the intrinsic ecosystems. Such contrasting observations provide new insights in a MAP and the intrinsic ecosystem driven divergent distribution pattern of *cbbL-* and *cbbM-*containing bacterial communities in arid soils. Several southern *cbbL-*containing generalist OTUs were identified as Bradyrhizobiaceae Phyllobacteriaceae and Nitrosomonadaceae, while several dominant northern *cbbM-*containing generalist OTUs were identified as Sterolibacteriaceae, Acidithiobacillaceae, Thioalkalispiraceae and Thiobacillaceae. The *Bradyrhizobium* in Bradyrhizobiaceae, *Mesorhizobium* in Phyllobacteriaceae and Nitrosomonadaceae are dominant autotrophic bacteria in crop soils (Yuan et al., 2012a,c; Wang et al., 2022). *Bradyrhizobium* and *Mesorhizobium* are also known as N_2_-fixing bacteria (Xu et al., 1995; Steenhoudt and Vanderleyden, 2000) and their inoculations have been shown to promote plant growth (Michiels et al., 1989; Molla et al., 2001). *Nitrosospira* in Nitrosomonadaceae is an important player in soil nitrification (Kowalchuk and Stephen, 2001). The general occurrence of these *cbbL* taxa in the southern region and previous studied crop soils suggests their important roles in soil C and N cycles in agricultural soils. Sterolibacteriaceae, Acidithiobacillaceae, Thioalkalispiraceae and Thiobacillaceae are involved in nitrogen and/or sulfur cycling (Aroca et al., 2007; Watanabe et al., 2014; Bazylinski et al., 2017), suggesting that these *cbbM* taxa might play an important role in soil functional processes (such as C and N and sulfur cycling) in the northern forest/grassland soils. The generalist taxa in the Chinese Loess Plateau were mainly chemolithoautotrophic bacteria, which might be due to the soil sampling depth 0–10 cm; a shallow soil layer, e.g., 0–1 cm, might get a higher proportion of phototrophic autotrophs such as Cyanobacteria (Zhao et al., 2018).

### 4.2 Microbial spatial structure in the Loess Plateau

A negative correlation of community similarity in *cbbL*- and *cbbM-*containing bacteria with increasing geographic distance indicates a spatial structure of autotrophic bacterial community. Moreover, the slopes of these relationships can differ, reflecting varying rates of species turnover in their habitats (Ranjard et al., 2013; Wang et al., 2017). Surprisingly, the slopes of *cbbL-* and *cbbM-*containing bacterial communities were similar and showed slight difference from that of total bacteria in the studied regions (Figure 4), suggesting their similar rates of species turnover in their habitats. Soil samples in the Loess Plateau had a significant environmental variability: a broad spatial scale, different habitats and management practices. The co-occurrence of abundant *cbbL* generalists in soils separated by great distances (0.3–391 km) and environmental heterogeneity suggests the action of dispersal processes in the *cbbL-*containing bacterial community. In contrast, the *cbbM-*containing generalist OTUs of the Loess Plateau were generally differentially selected by the northern and southern regions, suggests the limit dispersal processes and dominant environmental/ecosystem selection for the *cbbM-*containing bacterial community.

## 5 Conclusion

Our results highlighted the distribution patterns of autotrophic bacterial communities across the Chinese Loess Plateau soils, mainly driven by MAP and the related ecosystem types. The *cbbL-*containing generalist OTUs showed almost equal abundance in the southern and northern regions, while *cbbM-*containing bacterial taxa were more prevalent in the northern grassland/forest region. The co-occurrence of abundant *cbbL* generalists in the whole Loess Plateau, indicating the important action of dispersal processes. In contrast, the *cbbM-*containing generalist OTUs of the Loess Plateau were differentially selected by the northern and southern regions, suggesting the limited dispersal processes and dominant environmental/ecosystem selection. This study provides a new perspective on the distributions of autotrophic bacteria across different habitats, vegetation and climatic gradients, and the environmental drivers. This study also highlights that *cbbM* type autotrophic bacteria in soils might play an important role in natural ecosystems such as grassland and forest, which is needed further study.

## Data availability statement

The datasets presented in this study can be found in online repositories. The names of the repository/repositories and accession number(s) can be found in the article/Supplementary material.

## Author contributions

YW: Writing – original draft, Writing – review & editing. YH: Investigation, Writing – review & editing. QZ: Investigation, Writing – review & editing. DL: Investigation, Writing – review & editing. SA: Funding acquisition, Writing – review & editing.

## Footnotes

^1^ http://data.cma.cn/

^2^ http://ftp.ncbi.nih.gov/blast/db/

^3^ http://ftp.ncbi.nih.gov/blast/db/

^4^ http://www.ebi.ac.uk/ena/data/view/PRJEB58633
