# Supplementary material for: Biogeographic distribution of autotrophic bacteria was more affected by precipitation than by soil properties in an arid area
Source: Front Microbiol. 2023 Dec 20;14:1303469. doi: 10.3389/fmicb.2023.1303469 (PMC10761425; doi:10.3389/fmicb.2023.1303469)
Supplement: Supplementary file 1 [file Data_Sheet_1.pdf]

Supporting information

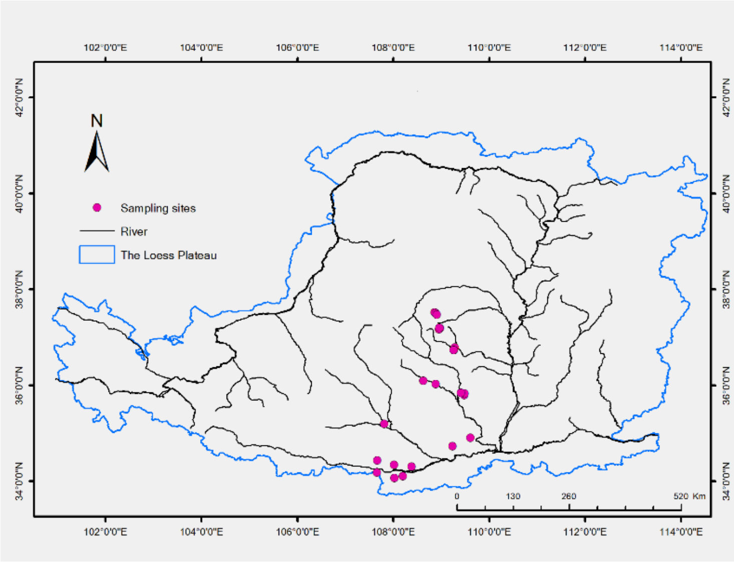

Supplementary Figure S1 The sampling sites (from north to south transect, pink color points) in the Loess Plateau of China.

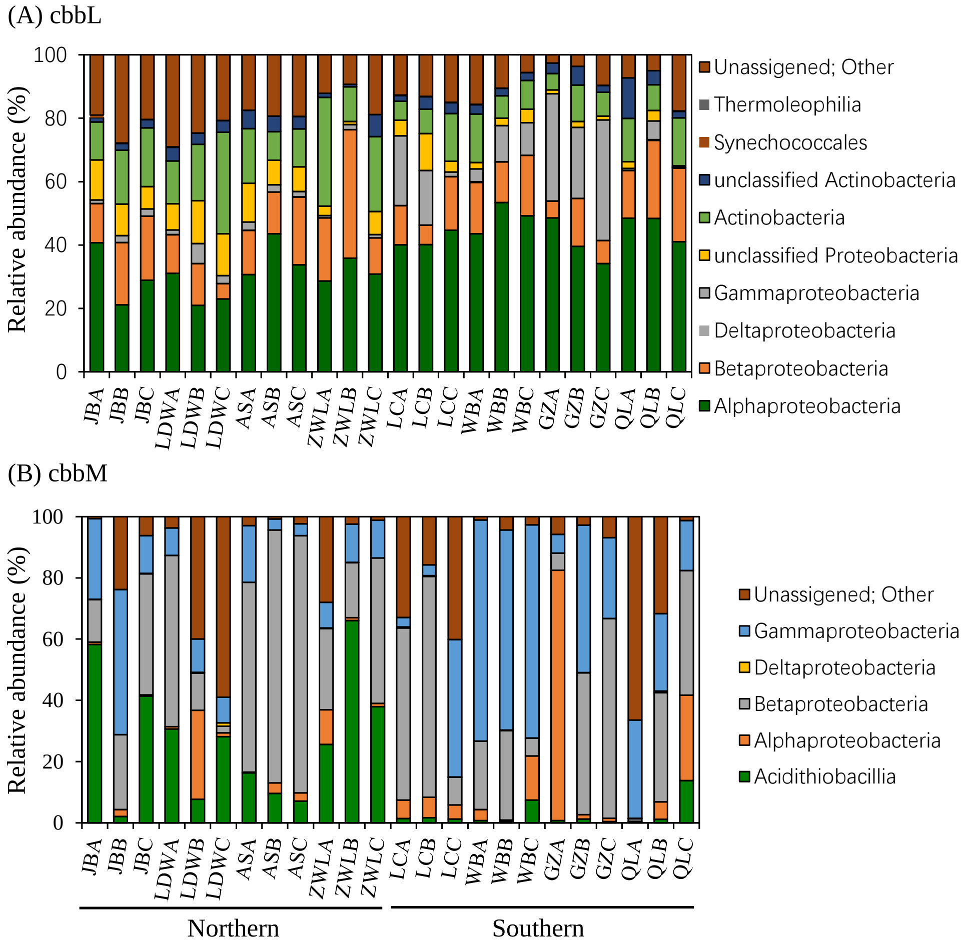

Supplementary Figure S2 Autotrophic bacterial taxa distributions in soils from the Chinese Loess Plateau. (A) *cbbL*-containing bacteria; (B) *cbbM*-containing bacteria. JB: Jingbian; LDW: Liandaowan; AS: Ansai; ZWL: Ziwuling; LC: Luochuan; WB: Weibei; GZ: Guanzhong; QL: Qinling, with 3 replicates (A, B, C), respectively.

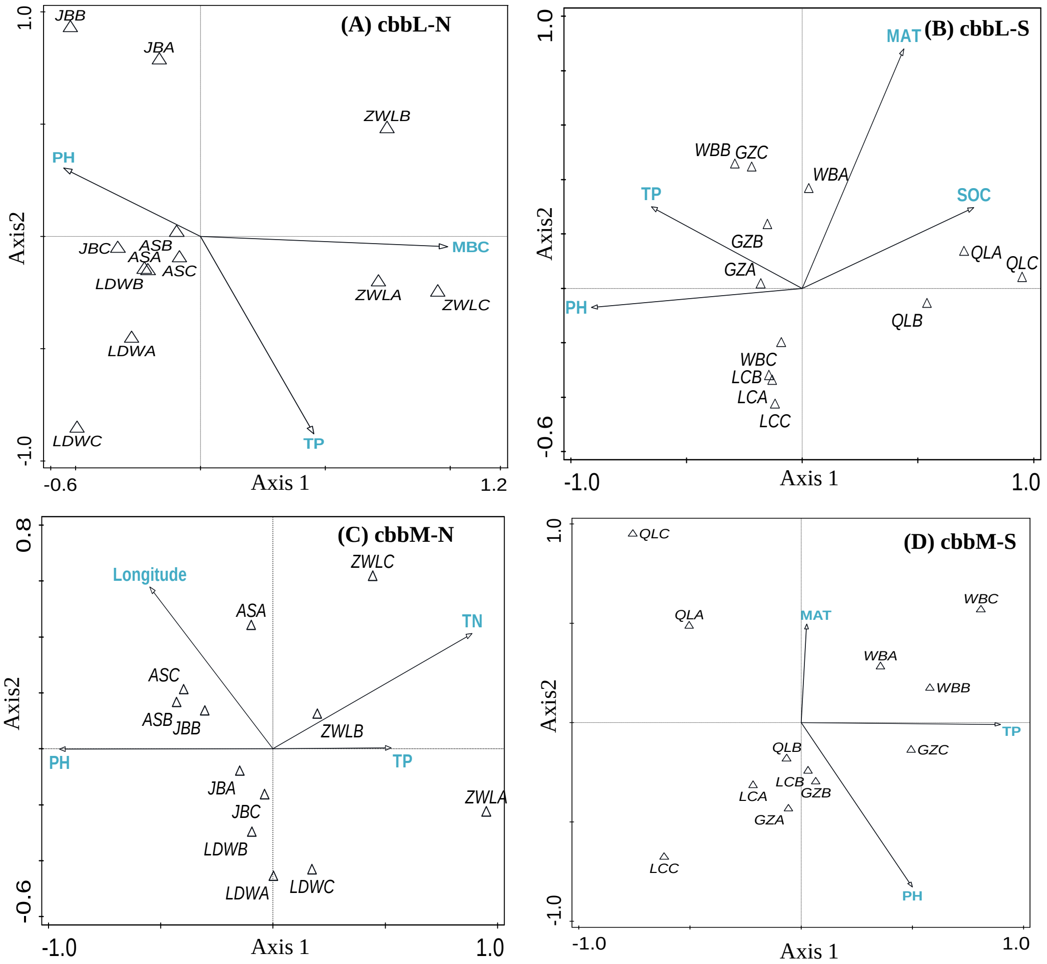

Supplementary Figure S3 Factors driving the β-diversity of soil *cbbL*- and *cbbM*-containing bacterial communities in the Chinese Loess Plateau. The *cbbL*-containing bacteria in northern (A) and southern (B) regions; *cbbM*-containing bacteria in northern (C) and southern (D) regions.

Supplementary Table S1 Spearman correlations among soil and climatic variables.

	pH	SOC	TN	TP	AV.P	Nmin	MBC	C/N	MAT	MAP	Altitude
SOC	-0.744**										
TN	-0.853**	0.909**									
TP	-0.683**	0.525**	0.577**								
AV.P	-0.747**	0.546**	0.636**	0.940**							
Nmin	-0.727**	0.505*	0.605**	0.930**	0.937**						
MBC	-0.380	0.709**	0.610**	0.289	0.328	0.263					
C/N	0.179	0.236	-0.024	-0.356	-0.409*	-0.458*	0.330				
MAT	-0.803**	0.619**	0.597**	0.732**	0.672**	0.668**	0.233	-0.162			
MAP	-0.845**	0.697**	0.795**	0.707**	0.740**	0.734**	0.456*	-0.235	0.830**		
Altitude	0.851**	-0.780**	-0.768**	-0.726**	-0.713**	-0.695**	-0.423*	0.066	-0.951**	-0.918**	
Longitude	0.198	-0.393	-0.383	0.038	0.041	0.039	-0.408*	-0.273	-0.195	-0.383	0.352

* $p < 0.05$ level.

** $p < 0.01$ level.

SOC: soil organic C; TN: total N; TP: total P; AV.P: available P; Nmin: the sum of ammonium and nitrate; MBC: microbial biomass C; MAT: mean annual temperature; MAP: mean annual precipitation.

Supplementary Table S2 Soil characteristics in the northern and southern Loess Plateau

Parameters	Northern	Southern	*p*
MAP	451±18‡	556±5	**<0.001**
MAT	9±0.1	11.6±0.4	**<0.001**
pH	8.88±0.06	7.93±0.22	**<0.001**
SOC	6.86±1.56	11.37±1.18	**0.02**
TN	0.56±0.12	1.14±0.09	**0.006**
TP	0.47±0.03	0.95±0.09	**<0.001**
AV.P	1.48±0.37	32.07±8.37	**<0.001**
Nmin	110.29±52.96	11.08±0.86	**<0.001**
MBC	205.51±51.49	174.83±25.7	0.30
C/N	9.91±0.51	11.45±0.85	0.18

‡Values are means of 12 soils ± standard error.

Supplementary Table S3 The alpha-diversity and families of *cbbL*- and *cbbM*-containing bacteria in the northern and southern Loess Plateau.

	Northern	Southern	*p*
cbbL			
Chao1	2651.3±272.4	2205.7±167.7	0.08
Shannon	8.42±0.23	7.96±0.23	0.11
Pseudonocardiaceae	16.48±2.41‡	8.42±1.11	**0.05**
Comamonadaceae	6.72±1.21	3.5±0.63	0.08
Nocardiaceae	0.94±0.17	0.69±0.12	0.12
Bradyrhizobiaceae	15.41±1.66	18.95±1.85	**0.05**
Phyllobacteriaceae	7.35±0.83	11.28±1.12	**0.02**
Burkholderiaceae	7.06±1.99	5.11±0.97	0.52
Rhodospirillaceae	2.69±0.41	4.2±0.57	**0.03**
Rhizobiaceae	0.28±0.09	0.7±0.09	**0.007**
Rhodobacteraceae	0.52±0.17	1.84±0.29	**0.002**
Nitrosomonadaceae	0±0	1.1±0.52	**<0.001**
cbbM			
Chao1	335.3±33.2	287.9±20.4	>0.1
Shannon	4.41±0.3	4.41±0.22	>0.1
Sterolibacteriaceae	25.88±6.44	9.95±4.38	**0.018**
Acidithiobacillaceae	27.56±5.93	2.52±1.17	**<0.001**
Thioalkalispiraceae	7.14±1.61	0.45±0.25	**<0.001**
Halothiobacillaceae	1.73±1.16	12.2±3.36	**0.003**
Chromatiaceae	3.63±2.97	11.74±4.75	0.35
Thiobacillaceae	12.58±5.59	13.39±5.68	0.64
Bradyrhizobiaceae	2.67±2.14	0.89±0.32	0.73
Rhodobacteraceae	0.62±0.26	1.27±0.78	0.84
Rhodospirillaceae	1.16±0.8	10.44±6.01	**0.006**
Comamonadaceae	0.17±0.13	5.57±3.75	**0.03**

‡Values are means of 12 soils ± standard error.

Supplementary Table S4 Variation in soil *cbbL*- and *cbbM*-containing bacterial community structure revealed by multi-response permutation procedure (MRPP) analysis.

	cbbL		*cbbM*	
	T	*p*	*T*	*p*
Northern vs. Southern	-7.80	***<0.001***	-7.85	***<0.001***

T, test statistic; more negative, more separation between communities.

Supplementary Table S5 Spearman correlations of soil chemical and microbial variables with *cbbL*-containing bacterial families in the northern and southern Loess Plateau.

	MAT	MAP	pH	MBC	TN	TP	SOC
Northern							
Pseudonocardiaceae	0.330	0.484	-0.650*	0.063	0.734**	0.545	0.671*
Comamonadaceae	-0.200	-0.382	0.371	-0.343	-0.189	-0.385	-0.252
Bradyrhizobiaceae	-0.302	-0.484	0.399	0.028	-0.601*	-0.133	-0.434
Phyllobacteriaceae	-0.126	0.126	-0.084	0.280	0.042	-0.077	0.070
Burkholderiaceae	-0.053	0.516	-0.210	0.804**	0.413	0.175	0.531
Rhodospirillaceae	-0.312	-0.263	0.140	0.140	-0.070	-0.343	-0.021
Nocardiaceae	0.221	-0.207	-0.084	-0.713**	-0.098	0.021	-0.252
Rhizobiaceae	0.000	0.505	-0.287	0.469	0.580*	0.329	.0601*
Rhodobacteraceae	-0.446	0.067	-0.140	0.448	0.210	-0.091	0.224
Southern							
Pseudonocardiaceae	0.224	0.231	-0.448	0.231	0.210	-0.531	0.252
Comamonadaceae	-0.154	-0.084	-0.329	0.154	-0.084	-0.091	-0.168
Bradyrhizobiaceae	0.531	0.301	-0.566	-0.035	0.657*	-0.259	0.462
Phyllobacteriaceae	-0.517	0.392	-0.140	-0.007	0.140	-0.455	-0.350
Burkholderiaceae	0.399	0.266	-0.804**	0.112	0.434	-0.559	0.441
Rhodospirillaceae	-0.133	-0.483	0.294	-0.091	-0.441	0.867**	-0.168
Nocardiaceae	-0.252	0.231	-0.154	-0.364	-0.021	0.224	-0.336
Rhizobiaceae	-0.175	0.280	0.070	-0.308	0.042	0.294	-0.294
Rhodobacteraceae	-0.084	-0.378	0.573	0.007	-0.441	0.762**	-0.126
Nitrosomonadaceae	-0.168	-0.427	0.741**	-0.196	-.594*	0.594*	-0.301

* $p < 0.05$ level.

** $p < 0.01$ level.

MAT: mean annual temperature; MAP: mean annual precipitation; SOC: soil organic C; TN: total N; TP: total P; MBC: microbial biomass C.

Supplementary Table S6 Spearman correlations of soil chemical and microbial variables with *cbbM*-containing bacterial families in the northern and southern Loess Plateau.

	MAT	MAP	pH	MBC	TN	TP	SOC
Northern							
Sterolibacteriaceae	-0.035	0.070	-0.042	0.329	-0.049	0.007	0.021
Acidithiobacillaceae	-0.102	0.200	-0.503	0.231	0.441	0.091	0.434
Thioalkalispiraceae	-0.270	-0.116	-0.329	0.175	0.259	-0.091	0.266
Halothiobacillaceae	-0.333	-0.663*	0.608*	-0.420	-0.685*	-0.427	-0.699*
Chromatiaceae	-0.011	-0.277	0.273	-0.378	-0.315	-0.182	-0.378
Thiobacillaceae	-0.646*	-0.540	0.587*	0.049	-0.413	-0.350	-0.364
Bradyrhizobiaceae	0.084	-0.428	0.657*	-0.706*	-0.538	-0.112	-0.573
Rhodobacteraceae	0.246	0.182	0.427	0.231	-0.350	-0.035	-0.231
Rhodospirillaceae	0.011	0.361	-0.154	0.413	0.189	0.252	0.154
Comamonadaceae	0.772**	0.828**	-0.448	0.168	0.549	0.634*	0.508
Southern							
Sterolibacteriaceae	-0.077	0.329	0.210	-0.154	-0.056	-0.182	0.042
Acidithiobacillaceae	-0.420	0.308	-0.315	-0.336	0.203	-0.049	-0.350
Thioalkalispiraceae	-0.664*	0.133	0.077	-0.077	-0.154	0.000	-0.434
Halothiobacillaceae	0.503	-0.091	-0.238	0.336	0.245	0.399	0.559
Chromatiaceae	0.049	-0.469	-0.021	-0.287	-0.245	0.734**	-0.098
Thiobacillaceae	-0.210	-0.441	-0.042	-0.434	-0.224	0.252	-0.301
Bradyrhizobiaceae	-0.755**	0.259	0.196	-0.098	-0.126	-0.154	-0.552
Rhodobacteraceae	-0.458	0.634*	-0.021	0.007	0.366	-0.542	-0.211
Rhodospirillaceae	-0.238	0.545	-0.140	-0.224	0.371	-0.007	-0.203
Comamonadaceae	-0.214	-0.196	0.581*	0.042	-0.515	-0.067	-0.259

* $p < 0.05$ level.

** $p < 0.01$ level.

MAT: mean annual temperature; MAP: mean annual precipitation; SOC: soil organic C; TN: total N; TP: total P; MBC: microbial biomass C.
